# Talking Convergence: Growing Evidence Links FOXP2 and Retinoic Acid in Shaping Speech-Related Motor Circuitry

**DOI:** 10.3389/fnins.2017.00019

**Published:** 2017-01-25

**Authors:** Moritz Negwer, Dirk Schubert

**Affiliations:** ^1^Max Planck Institute for PsycholinguisticsNijmegen, Netherlands; ^2^Department of Cognitive Neuroscience, Radboud University Medical Center, Donders Institute for Brain, Cognition, and BehaviourNijmegen, Netherlands

**Keywords:** FoxP2, retinoic acid, motor circuits, speech disorder, striatum, dopamine, RARB, globus pallidus

FOXP2 was the first identified monogenic cause of a speech disorder (for review see Graham et al., 2015). However, it remains to be answered how it affects the development of speech related neuronal circuits and what the relevant molecular pathways may be. Devanna et al. (2014) reported a direct interaction between FOXP2 and Retinoic Acid (RA), an important signaling molecule in brain development and neuronal differentiation, in a cell model. What could this finding mean for our understanding of a language-ready brain?

Mounting evidence from diverse fields such as linguistics, genetics, brain development, and cellular neurophysiology suggests that the brain's motor circuitry might be the key to the conundrum of language disorders. Human patients with FOXP2 mutations show prominent motor learning deficits at the root of their developmental verbal dyspraxia diagnosis, which is reflected in mouse models of Foxp2 loss (for review see Deriziotis and Fisher, 2013; Graham et al., 2015). Interestingly, in mice with dysregulated RA signaling, similar motor learning impairments have been found (for review see Duester, 2013). Retinoic acid has several prominent roles in brain development and function, for example as a diffusible signaling molecule that changes gene expression via nuclear retinoic acid receptors (RARs, RORs, and RXRs; for review see Maden, 2007).

Devanna et al. (2014) demonstrated a direct interaction between FOXP2 and RA signaling in a human neuroblastoma cell line (SH-S5Y5), in which RA exposure initiates differentiation into a dopaminergic-neuron like state (Korecka et al., 2013). When the researchers compared FOXP2 overexpression to RA treatment they found an interesting convergence: In both cases, the cells differentiated from their previously proliferative state into a more neuron-like state and showed elevated expression of RA signaling-related genes, specifically the receptor RARß.

A recent review of the same group (van Rhijn and Vernes, 2015) suggests that the motor-learning deficits found in carriers of FoxP2 mutations might be mediated by defects in RA signaling, also in humans. Interestingly, FoxP2 and RA signaling have also been found (separately) to be involved in vocal production learning in a songbird model (Denisenko-Nehrbass et al., 2000; Olson et al., 2011; Roeske et al., 2014; Wohlgemuth et al., 2014). Even though direct evidence of synergies between FoxP2 and RA signaling is still lacking beyond cell models, the hypothesis that they are parts of the same regulatory network (e.g. Benítez-Burraco and Boeckx, 2014; van Rhijn and Vernes, 2015) would fit well with a number of findings, some of which we will address in the following commentary.

## Striatal development: do FoxP2 and RA converge on specific cell types?

**Figure 1:**
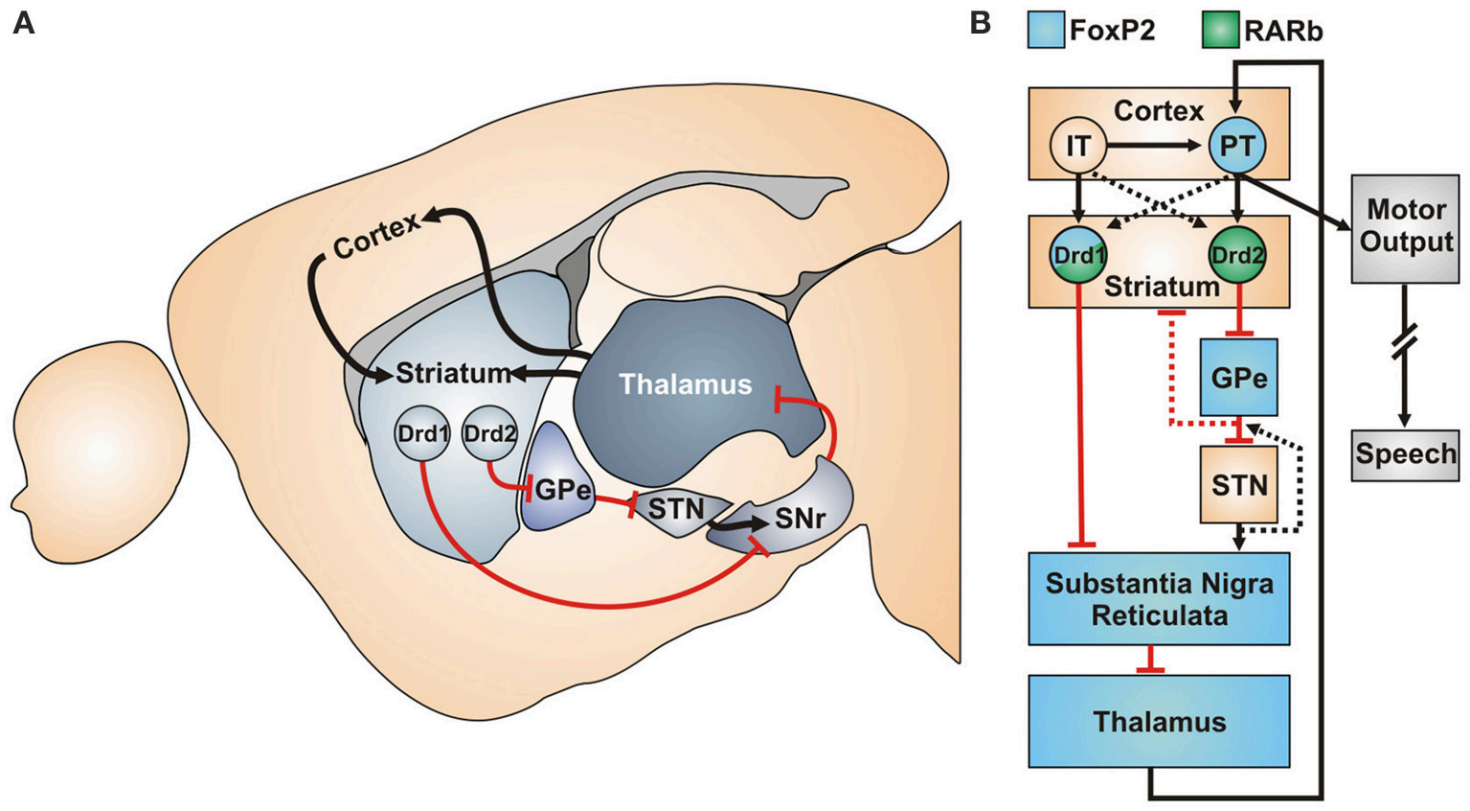
**Canonical cortico-basal-ganglia-thalamic circuit of the motor system (A)** Sagittal view of the mouse motor system with excitatory (black) and inhibitory projections (red) connections. **(B)** Functional model (based on Morita 2014) of the motor sequencing circuitry with FoxP2 and RARb expression. IT, Intratelencephalic neurons of the cortical layer V; PT, Pyramidal tract “motor neurons” of layer Vb; Drd1/Drd2, Striatal dopamine-receptor 1/2 expressing MSNs; GPe, Globus Pallidus External; STN, Subthalamic nucleus; SNr, Substantia nigra, pars reticulata.

The canonical cortico-basal-ganglia-thalamic motor circuit is thought to be crucial for the motor aspects of vocal production. Interestingly, in this canonical circuit FoxP2 (refers to the gene across vertebrate species) and RA receptor expression overlap in several regions (Figures 1A,B). An especially illustrative region in this regard is the striatum, a hub of the motor circuitry that is known to be involved in motor sequencing in mammals (Kravitz and Kreitzer, 2012; Friend and Kravitz, 2014) and also in vocal production learning in songbirds (Bolhuis et al., 2010; Scharff and Petri, 2011). Available data indicates both FoxP2 expression and RA in the development and function of the striatum (van Rhijn and Vernes, 2015).

In the developing rodent striatum, two layers of progenitor cells in the ventricular and subventricular zone (SVZ) generate the medium spiny neurons (MSNs), the main cell type of the striatum (Figure 1B). Early during striatal development, a mixed population of progenitor cells in the SVZ starts producing RA, concomitant with the start of FoxP2 and RARß expression in the same region (Li et al., 2000; Ferland et al., 2003; Molotkova et al., 2007; Liao et al., 2008; Garcia-Calero et al., 2015). In general, RA signaling is involved very early in the spatial patterning of the brain, starting with the formation of the telencephalic vesicle; however the precise extent has been controversially discussed (Siegenthaler et al., 2009; Chatzi et al., 2011, 2013; Duester, 2013). More importantly for motor circuit development, RA signaling is involved in the patterning of the striatum and striatonigral projections (Rataj-Baniowska et al., 2015) as well as migration of interneurons from the Ganglionic Eminences (that also produce MSNs) to the cortex (Crandall et al., 2011).

Within striatal MSNs, mouse studies imply an interesting convergence of FoxP2 and RA signaling onto a single cell population, namely D1R-MSNs (“direct pathway” neurons) of the dorsolateral striatum (Figure 1B). In mice lacking RARß, the progenitor population prematurely differentiates, specifically reducing the final cell numbers of D1R-MSNs (Rataj-Baniowska et al., 2015). Interestingly, RARβ was also one of the key RA receptors found to be affected in the human neuroblastoma cell study of Devanna et al. (2014). In mice lacking RARß, levels of the D1R-linked signal transduction protein Darpp-32 are greatly reduced (Liao et al., 2008). This is of interest not only because D1R-MSNs preferentially express FoxP2, but also because FoxP2 mutant mouse embryos likewise show strongly reduced Darpp-32 expression levels (Vernes et al., 2011, see supplementary material). One may therefore hypothesize that FoxP2 and RA signaling pathways converge in the development of the D1R-MSNs cell class in the dorsolateral striatum (Figure 1B), impacting the motor circuit's “direct pathway”.

If these hypotheses are applied to a computational model of motor sequencing (Morita et al., 2012; Morita, 2014), several possible consequences emerge. On the circuit-function level, FoxP2 and RA disruptions would impair D1R-MSNs function, and hence the “go” signal in motor control (Sippy et al., 2015, Figure 1B). Furthermore, FoxP2 mutations may affect inhibitory feedback to the striatum via a functionally defined population projecting from the Globus Pallidus Externa back to the striatum (Abdi et al., 2015; Dodson et al., 2015; Mallet et al., 2016), leading to additional defects in the “stop” motor control. Interestingly, a recent study found Foxp2 to regulate the development of the projection patterns of thalamic nuclei, another key integrative part of the motor circuitry (Ebisu et al., 2016).

## Downstream behavioral effects: do similar phenotypes imply convergent mechanisms?

In mice, Foxp2 loss impairs vocalizations after birth (e.g. Castellucci et al., 2016; Chen et al., 2016). However, mouse vocalizations are thought to lack a learning component, a key factor in human speech (see French and Fisher, 2014 for a review). In male zebra finches, a vocalization learning model, FoxP2 is expressed throughout the song system (Haesler et al., 2004, 2007; Scharff and Petri, 2011; Mendoza et al., 2015). Expression is especially high in the song-learning related area X of the striatum at the time when songs are acquired (Haesler et al., 2004; Thompson et al., 2013), and FoxP2 knockdown during this period disrupts social modulation of song variability (Murugan et al., 2013). Similar to the mouse striatal network, FoxP2 knockdown in the juvenile zebra finch leads to specific reductions of D1R and Darpp-32 expression in the dorsolateral striatum, specifically in area X (Denisenko-Nehrbass et al., 2000; Haesler et al., 2004, 2007; Olson et al., 2011; Murugan et al., 2013; Roeske et al., 2014). It should be noted that in the songbird striatum, D1R and D2R expression in MSNs overlap to a greater degree than in the mouse (Kubikova et al., 2010). Furthermore, striatal RA signaling plays an important role in the maintenance of birdsong: RA-producing enzymes are located in projection axons to area X, where the MSN-like neurons in turn express RA receptors. Functionally, RA may be important for balancing the input to area X neurons from vocal motor pathway (stereotyped song) versus anterior forebrain pathway (variable song) vocalization centers (Roeske et al., 2014; Wohlgemuth et al., 2014).

## FoxP2 and RA: synergy in a larger regulatory network for neuronal connectivity?

On the molecular level, the gene networks regulated by FoxP2 and RA signaling might overlap in specific parts of the motor circuitry. It is important to separate developmental effects from on-line functional effects: Developmental defects might lead to an impaired network setup (e.g. a smaller and miswired MSN population), while functional effects might impair the learning and function even of healthy networks (e.g. failure to balance synaptic inputs to MSNs). Furthermore, it should be noted that both FoxP2 and RA are broad regulators of large gene networks, also outside the motor circuitry.

In this context, a recent study is of particular interest: Chen et al. (2016) found that Foxp2 loss was associated with defects in dendritic spine formation in striatal neurons via de-repression of the autism risk gene Mef2C, leading to defects in corticostriatal connectivity. Research in a different murine cell model suggests that RA treatment indirectly interacts with Mef2C to specify a neuronal fate, via the transcription factor Sp1 (Elmi et al., 2007). It would therefore be highly interesting to see whether Foxp2, RA, Mef2C, and Sp1 are all part of the same regulatory network and influence the development of MSN connectivity.

Thus, with their finding of synergies between FOXP2 and RA in a cell model Devanna et al. (2014) made an important first step. Other studies provide indirect evidence that FoxP2 and RA signaling genes could be part of the same regulatory network and co-influence development and function of the speech-motor control circuits, especially the striatum. However, a direct proof beyond cell models is still lacking. Hence future studies in adequate animal models will have to confirm this synergy on the neuronal circuit level, also considering the more extensive regulatory molecular network affecting the development and functioning of speech-motor control circuits.

## Author contributions

DS and MN both reviewed the current literature, wrote the manuscript and prepared the figures with equal contributions.

### Conflict of interest statement

The authors declare that the research was conducted in the absence of any commercial or financial relationships that could be construed as a potential conflict of interest.
